# Evolution in Neuromodulation—The Differential Roles of Acetylcholine in Higher Order Association vs. Primary Visual Cortices

**DOI:** 10.3389/fncir.2018.00067

**Published:** 2018-08-28

**Authors:** Veronica C. Galvin, Amy F. T. Arnsten, Min Wang

**Affiliations:** Department of Neuroscience, Yale University, New Haven, CT, United States

**Keywords:** acetylcholine, neuromodulation, cholinergic, prefrontal cortex, V1, nicotinic, muscarinic

## Abstract

This review contrasts the neuromodulatory influences of acetylcholine (ACh) on the relatively conserved primary visual cortex (V1), compared to the newly evolved dorsolateral prefrontal association cortex (dlPFC). ACh is critical both for proper circuit development and organization, and for optimal functioning of mature systems in both cortical regions. ACh acts through both nicotinic and muscarinic receptors, which show very different expression profiles in V1 vs. dlPFC, and differing effects on neuronal firing. Cholinergic effects mediate attentional influences in V1, enhancing representation of incoming sensory stimuli. In dlPFC ACh plays a permissive role for network communication. ACh receptor expression and ACh actions in higher visual areas have an intermediate profile between V1 and dlPFC. This changing role of ACh modulation across association cortices may help to illuminate the particular susceptibility of PFC in cognitive disorders, and provide therapeutic targets to strengthen cognition.

## Introduction

Acetylcholine (ACh) plays many neuromodulatory roles in the developing and mature brain, including guiding neuronal development, determining arousal state, and modifying cortical responses to environmental events. This review will provide a brief summary of cholinergic anatomy and development, and contrast the roles of ACh in the mature primary visual cortex (V1) with those in visual association cortices, and in the newly evolved dorsolateral prefrontal cortex (dlPFC), the site of working memory circuits. Finally, we will discuss evidence for disruptions in normal cholinergic processing contributing to cognitive disorders, and how the organization and signaling mechanisms of dlPFC circuits may increase their sensitivity to cholinergic disruption.

## The cholinergic neurons in the basal forebrain

**Figure 1:**
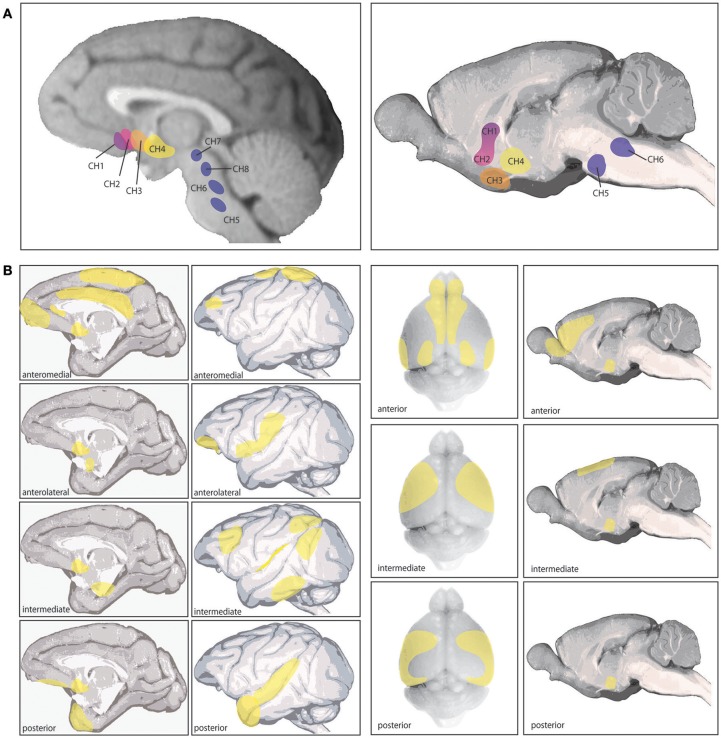
Cholinergic nuclei and cortical cholinergic projections as detailed in (Mesulam et al., 1983a,b; Luiten et al., 1987). **(A)** The left shows the 8 different cholinergic nuclei in primate brain, referred to as Ch1-8 (see text), and the right image shows the corresponding 6 cholinergic nuclei in rat brain. The 4 nuclei comprising the basal forebrain are Ch1-4. **(B)** The left shows the specific cortical projection patterns of the four distinct subsections of the Ch4 nucleus corresponding to the Nucleus Baysalis of Meynert (see text), based on those reported by Mesulam et al. (1983a). Ch4 anteromedial primarily projects to the midprincipalis, medial frontal pole, subcallosal gyrus, cingulate, dorsomedial motor cortex, and medal parietal cortex (areas 5 and 7). The anterolateral subsection projects to lateral area 12, frontal operculum, ventral S1, ventral posterior parietal cortex and the amygdala. The intermediate Ch4 region was further divided by Mesulam et al. (1983a) into a dorsal and ventral portion, which are combined here. The combined intermediate region innervates the ventrolateral orbital cortex, insula, periarcuate, posterior principalis, inferior parietal lobule, peristriate visual cortex, inferior temporal cortex, and parahippocampal regions. The posterior Ch4 subsection projects to the auditory association cortex and temporal pole. The right section shows the cortical projections in rodent arising from Ch4, based primarily on those reported by Luiten et al. (1987). In rodent these projections show a gradient pattern with considerable overlap between subsections. The anterior division projects to infralimbic, prelimbic, anterior cingulate, agranular insula, orbitofrontal in some animals, olfactory tubercle, piriform cortex, entorhinal cortex, occipital cortex in some animals, and motor and somatosensory cortex in some animals. This subsection appears to be a transition area between the intermediate region and the HDB (not pictured) which overlaps with all anterior Ch4 projections except amygdala, which HDB does not innervate. The intermediate Ch4 subsection innervates medial and lateral precentral cortex, motor cortex, somatosensory cortex in some animals, agranular insula, and perirhinal regions. The posterior section projects to motor, lateral precentral cortex, somatosensory cortex, temporal cortex, perirhinal cortex, and agranular insula and occipital cortex in some animals. In rodent, all Ch4 subdivisions strongly innervate amygdala. As this review focuses on cholinergic actions in different cortical areas and across species, these cortical projections are of key relevance.

In primates, ACh is synthesized by neurons in eight primary nuclei in the brainstem and basal forebrain. Four of these nuclei in the brainstem and midbrain (Ch5-8) innervate the thalamus, dopaminergic nuclei in the midbrain, interpeduncular brain stem nuclei, superior colliculus, and are implicated in arousal and sleep (Figure 1A; Steriade et al., 1988; Yeomans, 2012). The other four nuclei (Ch1-4) comprise the basal forebrain: the nucleus basalis of Meynert (NB), the horizontal limb of the diagonal band (DBh), the vertical limb of the diagonal band (DBv), and the medial septum (MS) (Figure 1A). Each of these nuclei show distinct cortical and subcortical projection patterns. The NB (Ch4) expresses the highest percentage of cholinergic neurons (>90%) and can be subdivided into four regions, innervating the entire cortical mantle and amygdala (Figure 1B). The MS (Ch1) and DBv (Ch2), expressing 10 and 70% cholinergic neurons respectively, providing innervation of the hippocampal formation and hypothalamus, and the DBh (Ch3) containing closer to 1% cholinergic neurons heavily innervates the olfactory bulb (Mesulam et al., 1983a).

Evidence suggests these cholinergic nuclei show significant differences between rodents and primates, most prominently in the specificity of afferent and efferent connections and in the proportions of GABAergic and other noncholinergic neurons to cholinergic cells (Mesulam and Mufson, 1984; Gritti et al., 1997; Zaborszky et al., 1997). Some of these differences may reflect an evolutionary trajectory in the expansion and specificity of PFC regions and the increasingly critical importance of ACh in PFC circuit function, where more precision in cortical cholinergic modulation is possible with a higher concentration of cholinergic neurons in basal forebrain and greater control over regional projection and release. There is some similarity in basal forebrain connectivity between rodent and monkey in projection patterns (Figures 1A,B), as well as innervation from cortical and subcortical regions, such as orbital PFC providing a major input to the nucleus basalis in both primates and mice (Mesulam and Mufson, 1984; Hu et al., 2016). However, as rodents do not have many regions of association cortex (e.g., dlPFC), comparisons of cortical circuits are necessarily limited.

Physiological recordings or calcium imaging from identified cholinergic cells in the basal forebrain of mice indicate high firing during waking and rapid eye movement sleep (Xu et al., 2015), and responses to meaningful sensory events related to movement and/or reward. For example, calcium imaging shows that cholinergic neurons fire to motor responses, e.g., licking a fluid reward in response to an auditory Go signal in mice (Harrison et al., 2016). Thus, they may integrate sensory, motor and value information. Cholinergic release in cortex in turn may enhance sensory processing of relevant cues, e.g., optogenetic stimulation of cholinergic neurons improved visual discrimination processing and enhanced the visual responses of neurons in V1 in mice (Pinto et al., 2013), and direct application of ACh in V1 of primate enhances neuronal responses to attended visual stimuli (Herrero et al., 2008). Older recordings from the nucleus basalis in monkeys were not able to identify cholinergic neurons, but nonetheless showed remarkably similar patterns to that seen in rodents, where neurons responded to the delivery of reward in a working memory task (Richardson and DeLong, 1986). These findings are consistent with the known inputs to the nucleus basalis from the orbital PFC in primates (Mesulam and Mufson, 1984), which provides flexible evaluation of reward value (e.g., Rudebeck et al., 2017). Similar projections from orbital PFC back to basal forebrain regions is also found in mouse (Hu et al., 2016). Although the roles of identified cholinergic neurons are just beginning to be understood, the information to date indicates important roles in sensory processing and goal-directed responding through their actions at cholinergic receptors in cortex.

ACh acts through both ionotropic nicotinic receptors and metabotropic muscarinic receptors expressed throughout the central nervous system. Nicotinic receptors are ion channels comprised of α (α2-α10) and β (β2-β4) subunits, forming a non-selective cation channel. Muscarinic receptors are metabotropic receptors, of which five functional subtypes have been identified, M1–M5. The M1, M3, and M5 receptors are coupled to Gα_q/11_, stimulating hydrolysis of phosphoinositol-diphosphate (PIP2) into inositol triphosphate (IP3) and diacetylglycerol (DAG), which release calcium from intracellular stores and activate PKC. The M2 and M4 receptors are coupled to Gα_i/o_, which inhibits adenylyl cyclase and reduces cAMP levels.

The cholinergic nuclei first appear and extend projections during embryonic stages of brain development, and may contribute to neural differentiation, migration, axon guidance, and local circuit formation and maturation across the cortex, as described in the following section.

## Acetylcholine in brain development

Studies examining receptor expression, ACh synthesis markers, alterations with genetic knock-down of receptor types, and physiological recordings all support a critical role for ACh throughout cortical and subcortical brain development to promote neuronal maturation, guide circuit formation, and refine synaptic connections.

There is evidence from both rodents and humans that nicotinic and muscarinic receptors are expressed in early embryonic development in cortical stem cells and progenitor cells, as early as E10 in mice and during the first trimester (4–12 weeks) in human embryonic development (Hellström-Lindahl et al., 1998; Atluri et al., 2001). While the precise role ACh plays at these receptors during these early stages in development is still unclear, there is evidence both receptor classes are functional and contribute to CNS development, as activation of muscarinic receptors expressed in neural precursors in the ventricular zone in rat promotes differentiation into neurons (Ma et al., 2000), and nicotinic receptors containing α3 or α7 subunits regulate the transition of GABA currents from excitatory to inhibitory during development in spinal cord, ciliary ganglion and mouse hippocampus (Liu et al., 2006). Nicotinic receptors passing calcium also may play a role in guiding developing nerve growth cones, as activation of nicotinic receptors initiated turning responses in nerve growth cones, and extracellular calcium was required for this response (Zheng et al., 1994). The role of ACh in early developmental cortical plasticity is also evident from studies showing that 6-OHDA depletion of norepinephrine and ACh abolishes plasticity in cat V1 from monocular deprivation during development (Bear and Singer, 1986), and this role of ACh in kitten V1 plasticity is primarily through actions on muscarinic receptors (Gu and Singer, 1993).

During these early stages there are other markers of ACh activity supporting a potential role for ACh during development of connections between subcortical and cortical regions. In particular, acetylcholinesterase (AChE), the enzyme which rapidly catalyzes the breakdown of ACh, is expressed in the developing primate brain during developmental windows where connections are being wired. During late embryonic development and the first few postnatal weeks in rhesus macaque development, AChE is transiently expressed in thalamic neuron axon terminals projecting to specific cortical areas, suggesting a potential contribution to guiding thalamic afferents to cortex (Kostovic and Rakic, 1984; Robertson et al., 1987; Mechawar and Descarries, 2001). This transient expression of AChE is also evident in fibers extending from both the mediodorsal thalamic nucleus (MD) and basal forebrain to the frontal lobes, suggesting a potential role in neuronal migration guidance or as a timing cue (Kostovic and Goldman-Rakic, 1983), though no causal studies have been yet conducted to test this correlation further, and it is unknown if this pattern is unique to PFC or also found for other cortical regions.

Interestingly, direct cholinergic fiber input from the basal forebrain to PFC and other cortical regions occurs during a partially overlapping time window in development, during perinatal and early postnatal periods in human. This early cholinergic innervation of the PFC from the basal forebrain shows laminar preferences, where most projections terminate in prospective layers III and IV (Kostović et al., 1988), where critical recurrent networks are forming and incoming thalamocortical afferents terminate, respectively. Through later development, these innervation patterns shift in a manner that varies across cortical regions. Initial innervation patterns during development are similar between primary sensory areas like V1 and those in PFC. These afferents first map onto thalamocortical and corticocortical circuit wiring layers. As the cortex matures, V1 retains dense ACh innervation in layers I, superficial II and layer IVc, poised to regulate corticocortical fibers and incoming thalamic inputs in primates. In contrast, ACh innervation in PFC loses layer IV reactivity, while layers III, V, and VI show AChE-reactive fibers, mapping onto lamina thought to be critical for recurrent PFC networks underlying working memory and for top down projections to other cortical and subcortical structures in primates (Kostović et al., 1988; Mesulam et al., 1992). Studies in cynomolgus monkeys staining PFC for choline acetyltransferase (ChAT), the enzyme that catalyzes the synthesis of ACh, show a similar though slightly varied laminar pattern, with high reactivity in layers I-III, and V (Lewis, 1991).

A key role for ACh in guiding circuit formation and maturation is also suggested by receptor expression patterns, which are timed to parallel the development of these AChE-rich thalamocortical projections and/or innervating ACh fibers from basal forebrain. In developing rat somatosensory cortex, nicotinic receptor expression is triggered by AChE-guided thalamic fiber ingrowth in the first week of postnatal life, as nicotinic α7 subunit mRNA expression begins ~1 day after thalamic fiber innervation at P0-P1 after there is also a transient expression in mRNA within corresponding thalamic nuclei, and unilateral electrolytic or cytochemical lesions result in marked reduction of α7 expression (Broide et al., 1996). Slice physiology experiments support a presynaptic expression pattern for nicotinic receptors in S1 of rat (Gil et al., 1997). In ferrets, these receptors mediate fast synaptic transmission within developing visual cortex, and play a functional role in circuit formation and remodeling within local cortical regions during these early developmental periods to set the stage for incoming sensory stimulus processing after birth, where the number of cells receiving fast cholinergic synaptic inputs increased with increasing thalamic afferent input, and the frequency of such events increased at eye opening (Roerig et al., 1997).

A variety of evidence from studies in mice indicates that cholinergic receptor mechanisms play a role in synapse formation. In hippocampus, nicotinic receptors containing β2 or α7 subunits contribute to spine development and synapse formation, and parallel findings may occur in V1, as suggested by findings from nicotinic β2, α7, or α5 subunit KO mice (Roerig et al., 1997; Bailey et al., 2012; Lozada et al., 2012a,b). For example, nicotinic β2 subunits are required for the spontaneous activity underlying early visual circuit development, which is driven by retinal waves before eye opening in mice (Ackman et al., 2012; Burbridge et al., 2014). Nicotinic β2 receptors may also be protective with age, as β2 KO mice also exhibit accelerated aging effects of both basal and apical dendritic loss in layer V neurons in ACC and, to a lesser extent, V1 (Konsolaki and Skaliora, 2015). Data also suggest muscarinic receptors contribute to refinement of circuit development in V1 in mice, where genetic knock-out of different muscarinic receptors alters retinotopic map development (Groleau et al., 2014).

ACh also plays a role in maturation of cortical projection neurons in layer VI through nicotinic receptors containing α4, α5, and β2 subunits (Kassam et al., 2008; Heath et al., 2010). Layer VI cortical neurons are highly sensitive to blockade of both α4β2 receptors and α4β2α5 during the first few weeks postnatally in rats, particularly those in PFC projecting to medial dorsal thalamus, supporting a critical role for these receptors in top down systems such as those for attention (Kassam et al., 2008). In mice, genetic knockout of the α5 subunit causes substantial alterations in the normal responsivity pattern of layer VI mPFC neurons, and alters dendritic morphological changes during key developmental windows, suggesting a critical role for α5 nicotinic receptors in PFC circuit refinement in mice as well (Bailey et al., 2012).

A role for these nicotinic receptors in the development of key PFC circuits is supported by additional genetic knock-out studies, where mice with no nicotinic β2 subunit expression had profound loss of dendritic length and spine density in layer III PFC pyramidal neurons, and no change in spine number or density in V1 (Ballesteros-Yáñez et al., 2010; Konsolaki and Skaliora, 2015). There is also evidence for a role of the nicotinic α7 subunit for proper synapse formation on spines, as deletion of this subunit caused altered glutamatergic synapse formation, and nicotine or ACh application in neonatal rat hippocampus or auditory cortex can initiate excitatory postsynaptic potentials (EPSPs) at previously silent synapses (Maggi et al., 2003; Metherate and Hsieh, 2003; Lozada et al., 2012a). The PFC is known to exhibit increased dendritic complexity and spine density across evolution, which may be a key factor in susceptibility of this region to deficits in spine formation. The increase in synaptic spine density in PFC, and in excitatory communication between pyramidal neurons relying on synapses on spines, may underlie greater alterations in these circuits following disruptions in cholinergic signaling and receptor expression (Elston et al., 2006; Gilman et al., 2017).

ACh actions in early development may also contribute to formation and maturation of other neuromodulatory systems, as muscarinic receptor activation during early stages regulates the development of ascending dopamine systems in the striatum (Zhang et al., 2002). Additionally, nicotinic receptor subunits show changes in expression profiles in midbrain dopamine neurons through development, and have been shown to modulate both noradrenergic (NE) during hippocampal development (Leslie et al., 2002), and both NE and dopamine (DA) release in the mature brain in rodents (Liskowsky and Potter, 1985; Léna et al., 1999; Azam et al., 2007).

The developmental actions of ACh support a key role in axonal guidance, circuit formation, circuit refinement, excitatory and inhibitory balance, as well as for development of other neuromodulatory systems. These developmental roles shift into a key role in mature cortical functioning for enhancing encoding of important sensory information, for activating higher order PFC circuits, and for top down attentional processes across the cortical mantle.

## Mature cortex

**Figure 2:**
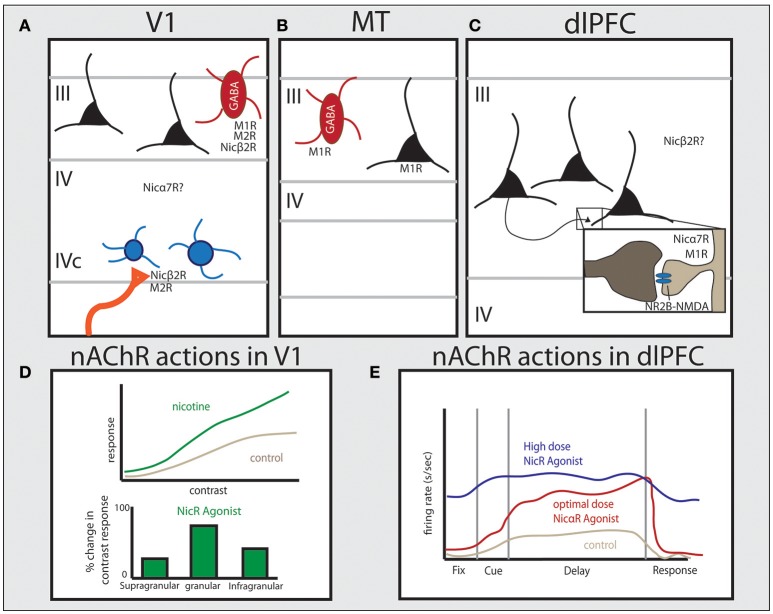
Distribution of cholinergic receptors across cortical regions in primate and their physiological functions. Studies in primate have shown unique patterns of receptor expression for both nicotinic and muscarinic cholinergic receptors between cortical areas. **(A)** In V1 muscarinic receptors are expressed by GABAergic cells (M1 and M2) or presynaptically (M2), and nicotinic β2-containing receptors are predominantly expressed presynaptically on thalamocortical terminals. Nicotinic α7 receptors are also expressed in V1 but their precise localization is still unclear. **(B)** In area MT, M1 receptors are expressed by both GABAergic cells and the majority of excitatory pyramidal cells. **(C)** In layer III of dlPFC, NMDAR-NR2B are found within the PSD of glutamate spine synapses. Muscarinic receptors are found within or near the PSD on spines (M1) or presynaptically on presumed ACh terminals (M2). Nicotinic α7 receptors are also found within or near the PSD on spines in primate layer III dlPFC. The specific distribution pattern of α4β2 receptors in primate PFC is unknown. **(D)** The effect of nicotinic receptor activation in monkey and tree shrew V1, based on data from Disney et al. (2007) and Bhattacharyya et al. (2012), showing that activation of nicotinic receptors significantly increases gain response in layer IV, but not in other layers. **(E)** Data from dlPFC based on Yang et al. (2013) showing that nicotinic α7 receptor stimulation enhances Delay cell persistent firing at optimal doses. See text for more details.

Across the mature cortex, ACh plays a prominent role in processing of sensory information and in cognitive processes in the adult brain. The correspondence of basal forebrain cholinergic neurons with sleep-wake states, where basal forebrain neurons have very low activity during deep sleep and high activity levels during REM sleep and wake states, indicates an important role for baseline cholinergic tone for many conscious and attention-related neural functions (Jones, 1993; Xu et al., 2015). Within the waking state, ACh can optimize cortical processing through actions at both nicotinic and muscarinic receptors. As described below, ACh can modulate sensory tuning in V1, both by enhancing incoming thalamocortical signals and reducing corticocortical inputs to primary sensory regions through actions on GABAergic interneurons. Cholinergic receptor expression shifts across visual association areas to increase direct actions on excitatory neurons (Figures 2A–C). In higher order association cortices, data from our lab suggests ACh plays a key permissive role for NMDA receptor glutamate actions that are needed for working memory and top down attentional control in dlPFC.

### Ach in primary visual area V1

Studies identifying specific receptor distributions and localization, along with physiological recordings manipulating ACh levels or receptors, have provided a scaffold for understanding ACh actions in V1. While this section includes data from rodent, tree shrew, cat, nonhuman primate and human, there is some evidence of species differences, suggesting experiments of cholinergic influence on V1 activity in rodents may not directly translate to primate. For example, muscarinic M1 receptor expression profiles across cell types in V1 show significant species differences, where in human, rhesus macaque, and guinea pig, 74–85% of parvalbumin-expressing GABAergic interneurons also express M1Rs, compared to 27% in rats (Disney and Reynolds, 2014). Species have been labeled to clarify differences where possible, though much more work needs to be done to determine where cholinergic receptor expression and ACh actions overlap and where they diverge across species and regions. For a more thorough review of species differences in the cholinergic system in the brain, see (Coppola and Disney, 2018).

In mature human cortex, the density of cholinergic innervation is graded, with the least innervation of V1, and increasing innervation through downstream visual areas (e.g., areas 20 and 21) (Mesulam et al., 1992). This pattern is in contrast to studies in rat, which generally show similar cholinergic innervation of V1 and other cortical regions (Lysakowski et al., 1989). In humans, primary visual area V1 (Brodmann area 17) has a distinctive pattern of cholinergic innervation, with a relatively higher density innervating superficial layers I and II, and layer IVc (Mesulam et al., 1992), mapping onto the critical lamina for corticocortical afferents and incoming sensory information from thalamic relay nuclei. This specific laminar organization is similar to rodent, and in human also matches the patterns of nicotinic and muscarinic receptor expression in this cortical region.

In primate V1, nicotinic β2 subunits are expressed presynaptically by 76% of thalamocortical axons targeting dendritic spines in layer IVc, but show very low expression on intrinsic neurons within V1, with expression within V1 primarily found on GABAergic interneurons (Figure 2A; Disney et al., 2007). These nicotinic receptors contribute to enhancing visual input from thalamic relay nuclei, as application of nicotine in V1 *in vivo* increased gain response in layer IVc neurons to visual stimuli, with a monotonic response pattern across a wide range of doses (1–160 nV) (Disney et al., 2007). This is supported by studies in V1 of the tree shrew and rodent, where nicotinic agonists strongly enhance contrast sensitivity within the granular input layer (Figure 2D; Bhattacharyya et al., 2012; Soma et al., 2013). Consistent with β2-containing receptors being expressed in GABAergic neurons elsewhere in V1, nicotine application outside of layer IVc in both more superficial (layers II and III) as well as deeper (layer V) neurons suppresses visual responses in V1 in monkey (Disney et al., 2007). Taken together, these data show that ACh in V1 is positioned to both amplify incoming sensory information while suppressing corticocortical processing (Disney et al., 2007), similar to rodent piriform cortex and hippocampus (Hasselmo and Bower, 1992; Hasselmo and Schnell, 1994; Hasselmo, 1995). Recent data suggests the developmental transition for V1 nAChRs away from regulating circuit wiring to dynamically modulate circuit dynamics in adulthood without significant plasticity is due to molecular modulation of these receptors by the endogenous prototoxin that binds to nAChRs, lynx1 (Morishita et al., 2010).

M1 and M2 receptors are also highly expressed on GABAergic interneurons in primate V1 (Disney et al., 2006). Parvalbumin-expressing (PV) interneurons comprise roughly 75% of the inhibitory population in V1, and as many as 87% of these PV neurons contain M1R protein, while 25% express M2 protein (Figure 2A; Disney and Aoki, 2008). In superficial layers, muscarinic receptor expression is localized to the soma in GABAergic neurons (Disney et al., 2006), supporting a role for ACh actions through muscarinic receptors in suppression of corticocortical projections in favor of enhancing sensory inputs. Presynaptic M2 labeling is predominantly in layer IVa and IVc, with particularly strong and homogeneous expression in sublayer IVcβ in monkey. These M2R expression patterns map closely with geniculocortical parvocellular projections as well as cholinergic terminals, indicating these receptors are poised to regulate incoming color and fine detail sensory information from thalamic nuclei on both excitatory presumed glutamatergic axons, as well as cholinergic afferents from the basal forebrain (Mrzljak et al., 1993, 1996). Genetic KO studies in mice support a key role for both M1-like and M2-like muscarinic receptors in V1 circuit refinement, as these genetic alterations cause disruptions in visual field size (Groleau et al., 2014).

Physiological recordings in primates show that ACh activation of muscarinic receptors in V1 produces a consistent enhancement in neuronal activity to attended visual stimuli in primates through actions on muscarinic receptors (Herrero et al., 2008) and improves contrast sensitivity and orientation tuning in tree shrew V1 (Bhattacharyya et al., 2012). ACh application in cat V1 show a mix of either enhancement or reduction in neuronal responses to stimuli, which may depend on the subpopulation or a combination of pyramidal and GABAergic interneuron activation for tuning responses (Sato et al., 1987; Murphy and Sillito, 1991). The cellular bases for muscarinic actions may involve increased pyramidal neuron excitability, e.g., through closing postsynaptic K^+^ channels, as reviewed in Thiele (2013), while the improved contrast sensitivity and orientation tuning may involve enhanced GABA actions (Disney et al., 2012). Cholinergic actions on GABAergic interneurons is also supported by recordings and ACh iontophoretic application in anesthetized cats, which is likely mediated by muscarinic receptor activation, as these effects were blocked by application of the muscarinic antagonist scopolamine (Müller and Singer, 1989). For a more in-depth review of muscarinic actions in V1, see (Groleau et al., 2015).

### Ach in higher visual areas—V2, V4, MT

Cholinergic modulation of neuronal activity continues through extrastriate higher order visual areas through both nicotinic and muscarinic receptor actions (Reynolds et al., 2000; Womelsdorf et al., 2006). Higher visual areas show strong modulation from visual attention (Luck et al., 1997; Reynolds et al., 2000; Womelsdorf et al., 2006), and ACh mechanisms underlying attentional modulation is supported by lesion studies, where lesions to the basal forebrain in monkeys impaired performance of a covert-attention shifting task that relies on parietal visual association cortex (Voytko et al., 1994). Although this lesion study could not distinguish between noncholinergic and cholinergic neurons in the basal forebrain, it is consistent with studies in rats showing that ACh is important for the attention functions of parietal cortex (Bucci et al., 1998; Chiba et al., 1999). Additionally, infusions of muscarinic receptor antagonists into intraparietal cortex in primates significantly reduced validity and alerting in a cued target detection task (Davidson and Marrocco, 2000). The different effects of cholinergic activity in visual association cortices may in part be due to changes in cholinergic receptor expression across higher visual areas.

One carefully documented difference in higher visual areas compared to V1 is in regard to muscarinic receptor expression, where there is a greater number of excitatory glutamatergic pyramidal neurons expressing M1 and M2 receptors in V2 than V1 in primate (Disney et al., 2006). These receptor data indicate a shifting pattern of M1 expression from primarily GABAergic in primary sensory cortices to primarily pyramidal neuron in higher order association cortices (Figures 2A–C) (Mrzljak et al., 1993; Disney et al., 2006). This change may underlie the effects of attentional modulation on neuronal activity in higher visual areas in primates, where attention increases firing rates and sensitivity to stimuli in V4 (Reynolds et al., 2000). Visual attention also enhances neuronal activity to relevant targets between competing stimuli within the reference field of neurons in both V2 and V4, where reference fields become increasingly larger compared to V1 (Luck et al., 1997; Reynolds et al., 2000). Attention also differentially alters correlated activity patterns in V4 compared to V1 of primate, where across lamina attentional modulation shows an inverted U-shaped pattern on increasing correlated variability in V4 compared to the U-shaped profile on correlated variability in V1 (Hansen et al., 2012; Nandy et al., 2017). This distinct effect of attention between superficial, granular, and deep cortical layers is also found with ACh application and receptor actions in V1 of tree shrew (Bhattacharyya et al., 2012) and mouse (Verhoog et al., 2016) compared to other cortical regions, supporting ACh release with attention.

Differences in cholinergic mechanisms between V1 and higher order visual cortices are particularly striking when comparing muscarinic influences in V1 vs. area MT. While muscarinic influences in V1 are primarily on GABAergic interneurons where they suppress sensory processing (Disney et al., 2012), in area MT M1 receptors are expressed on the majority of pyramidal neurons as well as PV-expressing neurons (Figure 2B; Disney et al., 2014), and ACh application substantially increased neuronal firing (Thiele et al., 2012). Although it is not known whether ACh excitatory effects were through muscarinic and/or nicotinic receptors in MT, cholinergic excitation was distinct from GABAeric mechanisms. Thus, although both ACh and the GABA_A_ receptor blocker, gabazine, increased neuronal firing rates in MT, only ACh application enhanced neuronal discrimination abilities and reduced intrinsic activity correlations, while gabazine reduced directional tuning by widening the tuning width (Thiele et al., 2012). The effects of ACh application in area MT mimic what is seen with spatial attention, supporting the release of ACh as a mechanism for attentional changes to neuronal properties (Womelsdorf et al., 2006; Mitchell et al., 2007, 2009). Human pharmacological data are generally consistent with a greater role for ACh in visual association cortices than in earlier cortical areas, as enhancing ACh modulation has a greater effect on voluntary attentional modulation than on bottom-up salient cue detection (Rokem et al., 2010). These findings are consistent with the important role of acetylcholine in attentional mechanisms across cortical regions, as well as for key higher order cognitive functions such as working memory in dlPFC, as described in the next section.

### Ach in dlPFC

The PFC is critical for executive functioning, working memory, and top-down regulation of emotions, actions and attention. The dlPFC in particular is critical for spatial working memory, or the ability to hold in mind spatial information over a delay in the absence of continued sensory input (Goldman and Rosvold, 1970; Goldman et al., 1971). The cellular organization underlying spatial working memory has been the most extensively studied of PFC functions. Thus, this section will focus on cholinergic actions within working memory circuitry in dlPFC specifically.

Electrophysiological recordings from dlPFC in monkeys identified neurons that show persistent firing across the delay period during a spatial working memory task (Funahashi et al., 1989). These neurons, termed “Delay” cells, exhibit persistent firing for a specific (or “preferred”) spatial location, and thus are thought to represent the cellular basis for spatial working memory. Evidence from anatomical studies indicates Delay cell persistent firing relies on pyramidal neurons in deep layer III of dlPFC, which have extensive horizontal projections, allowing for recurrent excitation between and within columns of neurons with similar preferred directions (Kritzer and Goldman-Rakic, 1995). The spatial selectivity of this activity, where Delay cells show elevated activity for particular preferred spatial locations, is tuned through lateral inhibition primarily from fast-spiking PV GABAergic interneurons (Goldman-Rakic and Schwartz, 1982; Goldman-Rakic, 1995; Kritzer and Goldman-Rakic, 1995; Rao et al., 1999, 2000; González-Burgos et al., 2000; Constantinidis and Goldman-Rakic, 2002).

The PFC is highly dependent on modulatory state (e.g., Brozoski et al., 1979; Arnsten et al., 2012), and recent studies emphasize the critical role of ACh. For example, cholinergic depletion of the PFC causes working memory deficits in monkeys equivalent to total tissue ablation (Croxson et al., 2011). A critical role for ACh in working memory is also supported by receptor distribution and physiology data. Recurrent excitation underlying persistent delay-related activity relies on NMDA receptor (NMDAR)-containing synapses on dendritic spines in deep layer III of dlPFC (Figure 2C), containing both NR2A and NR2B subunits (Wang et al., 2013). In classic circuits, NMDARs are typically localized within the postsynaptic density (PSD) with AMPA receptors (AMPARs), which provide critical membrane depolarization for relieving the magnesium block of NMDARs and permitting activation of NMDARs in concert with glutamate binding (Edmonds et al., 1995). Interestingly, the layer III dlPFC circuits underlying working memory do not show this same dependence on AMPARs, as AMPAR blockade has only a minimal reducing effect on Delay cell firing (Wang et al., 2013). Instead, ACh actions at nicotinic α7 receptors have been found to play the critical permissive role for NMDAR activation in these dlPFC circuits, where iontophoretic application of α7 antagonists cause marked reductions in persistent activity, and prevents the enhancing effects of direct NMDA application (Yang et al., 2013). Conversely, iontophoretic stimulation of nα7 receptors in dlPFC greatly enhances delay-related firing (Figure 2E). The expression pattern of α7 in layer III of dlPFC in primates is consistent with this physiological profile, where nicotinic α7 receptors are found within the postsynaptic density of glutamatergic synapses on spines (Figure 2C; Yang et al., 2013). Thus arousal state, as mediated by cholinergic stimulation, plays a critical permissive role for allowing NMDAR network connectivity in the primate dlPFC.

The permissive role of nicotinic α7 receptors in dlPFC for working memory contrasts with that in V1, where general nicotinic antagonists have no consistent role in attentional modulation (Herrero et al., 2008). There is some evidence that α7 receptors may play a permissive role for NMDAR activation in developing auditory cortex, where dual immunolabeling and electron microscopy experiments show a subset of synapses remaining α7 dependent (absent of AMPAR) into adulthood in rats (~25%) (Levy and Aoki, 2002). A variation on this theme has been seen in mouse hippocampus, where acetylcholine release during transition to wakefulness acts on astrocytic nicotinic α7 receptors to release D-serine and co-stimulate NMDARs (Papouin et al., 2017). It is not known if the same permissive role of ACh exists for other circuits in PFC underlying other cognitive behaviors, though PFC cholinergic depletion does not impact many cognitive functions in primates and the critical role of ACh may be particular to visual and attention-based functions (Croxson et al., 2011).

There appear to be species differences in nicotinic mechanisms in PFC, which may relate to the large evolutionary changes in PFC across species. Deep layer III PFC circuits are the most expanded across evolution, and layer III pyramidal neurons show substantial differences compared to PFC layer VI in primates, a difference not seen between layers in mouse (Amatrudo et al., 2012; Gilman et al., 2017). Thus, it is unclear whether these same circuits function in rodent PFC, though physiological recordings in mouse suggest a small proportion of layer II/III neurons express α7 postsynaptically and these receptors contribute to depolarization (Poorthuis et al., 2013). The question of whether persistent activity for working memory relies on N2RB-containing NMDARs in rodents is uncertain, though recent evidence suggests either this may be an evolutionarily distinct mechanism unique to primates (McQuail et al., 2016), or this recurrent circuitry instead resides in the much larger layer V in rodents (Wang et al., 2008).

In both rodents and primates, the PFC is also critical for top down attentional control, by guiding attention using goals held in mind. Additionally, it is known attentional control relies on PFC integrity, as dysfunction of PFC is associated with significant attentional deficits (Berry et al., 2014; Fernández-Jaén et al., 2015). Attention can be driven by bottom-up processes from salient visual stimuli in the environment, or from PFC top-down processes directing attentional resources (Buschman and Miller, 2007), the former of which may not require PFC ACh release at all, and latter of which mediates cholinergic release in other cortical areas (Nelson et al., 2005; Rokem et al., 2010). In rats, rapid cued ACh release is needed for accurate detection of sensory events, in addition to baseline cholinergic tone needed for PFC function (Parikh et al., 2007; Croxson et al., 2011).

Conversely, attentional control is needed for working memory tasks, to protect the contents of working memory and maintain attention on the task. For example, dlPFC Delay cell persistent activity is reduced (but not eliminated) when a distracting stimulus is presented during the delay. This reduction in activity can be blocked by iontophoretic application of a nicotinic α4β2 receptor agonist (Sun et al., 2017). A role for these receptors in sustained attention is also supported by studies in rodents, where attentional deficits in α4β2 KO mice are alleviated after lentiviral re-expression of α4β2 exclusively in PFC (Guillem et al., 2011). Nicotinic α4β2 receptor stimulation also enhances the firing of “Fixation cells” in the primate dlPFC, neurons which sustain firing throughout the duration of each working memory trial. The firing of Fixation cells can be significantly reduced or enhanced by application of a α4β2 agonist or antagonist, respectively, consistent with a role in sustained attention (Sun et al., 2017). The subcellular locations of α4β2 receptors on neurons in primate dlPFC are not known, but rodent studies suggest they may enhance catecholamine release (Kennett et al., 2012), and thus may have some of their effects through indirect beneficial actions. In mouse there is indirect evidence of postsynaptic α7 expression on pyramidal neurons, as modulation of synchronized cortical up states by α4β2 receptors is mediated through GABA_B_ receptors, but α7 receptor contributions are not (Sigalas et al., 2015).

Muscarinic mechanisms also play a large role in dlPFC function. There has been a longstanding history showing that blockade of muscarinic receptors with scopolamine impairs working memory in monkeys (e.g., Bartus and Johnson, 1976). Muscarinic M1 receptors are primarily localized postsynaptically on excitatory spines in PFC, while M2 receptors maintain their predominantly presynaptic expression profile in the prefrontal cortex (Figure 2; Mrzljak et al., 1993). This contrasts with mouse mPFC, where M1 expression is primarily on PV interneurons, similar to V1 (Douglas et al., 2002; Yi et al., 2014), though whole cell recordings in rat PFC suggest minimal actions on PV interneurons, and greater ACh influence on CCK+ interneurons and peptide-containing GABAergic cells (Kawaguchi, 1997). In monkey dlPFC, muscarinic receptor stimulation is needed to maintain the neural representations that underlie working memory. Iontophoresis of the muscarinic antagonist, scopolamine, markedly abolished neural representations of rules in a working memory task at a dose that only moderately reduced activity in V1 (Herrero et al., 2008; Major et al., 2015). However, the muscarinic response in dlPFC is complex, as nonspecific excitation of muscarinic receptors in dlPFC also eroded rule selectivity (Major et al., 2018), indicating a need for precise levels and patterns of muscarinic receptor engagement. The role of muscarinic receptor mechanisms in monkey dlPFC, including the contributions of M1R vs. M2R stimulation, is an area of ongoing research, where preliminary data indicate a major contribution of M1R to network function (Galvin, Wang, and Arnsten, unpublished).

The key role of Ach in PFC function may also extend to PFC coordination of ACh actions across cortex, as described below.

## Cholinergic coordination across cortical regions

Cholinergic actions in PFC may have reverberating effects throughout cortex, as PFC regulates the basal forebrain and in turn, cholinergic release in other cortical areas.

The PFC shows extensive projections back to the nuclei of the basal forebrain in both rat (Gaykema et al., 1991; Zaborszky et al., 1997) and primate, particularly the limbic PFC regions such as the orbital and medial PFC, which receives the densest cholinergic input (Mesulam et al., 1992; Ghashghaei and Barbas, 2001). These PFC projections can thus regulate the release of acetylcholine in other brain areas to guide behavior (Nelson et al., 2005), particularly visual attention and visual signal enhancement (Gritton et al., 2016). For example, activity in rodent PFC is able to stimulate coordinated ACh release in parietal cortex, but parietal is not able to do the same in PFC (Bucci et al., 1998; Moore and Armstrong, 2003; Nelson et al., 2005; Parikh et al., 2007; Pinto et al., 2013). Thus, impaired cholinergic regulation in PFC may have widespread ramifications across cortex, which may contribute to cognitive disorders.

## Neurological diseases associated with cholinergic dysfunction and potential interpretations

Several psychiatric disorders are associated with changes in cholinergic genes or function, and show alterations in high order sensory processing as well as significant PFC impairments. Some of the differences outlined above may help us understand how alterations to cholinergic signaling can cause very different effects depending on the age of insult and component of ACh signaling implicated, based on what we know about the different ways ACh influences circuitry in primary sensory compared to higher order association cortices. Understanding these differential effects can improve our understanding of symptom profiles as well as inform optimal therapeutic development.

While initial studies found dysfunction within the DA system in schizophrenia, acetylcholine receptors, and ACh signaling also appear to play a role in the disease. Schizophrenia is a developmental disorder characterized by three main clusters of symptoms: positive, such as hallucinations and delusions, negative, such as emotional blunting and alogia, and cognitive, such as impairments in working memory, abstraction, attention, and executive functioning. DA dysregulation is evident in positive symptoms, and current antipsychotic drugs target D2 receptors, but no current medications exist for treatment of negative or cognitive symptoms. Genetic wide association studies (GWAS) have found associations within the gene locus for the nicotinic α7 subunit (Bakanidze et al., 2013), and there is evidence of reduced α7 protein in PFC of patients (Guan et al., 1999). As this receptor is important in both primary sensory V1 and auditory circuit development, as well as critical for working memory circuitry in dlPFC, the disruption in brain function from altered α7 receptors may cause critical changes in early neural development many years prior to symptom expression (Reichenberg et al., 2010), as well as potentially weakening dlPFC network connectivity in mature circuits (Yang et al., 2013). Agonists for nicotinic α7 receptors have been developed for potential use to treat cognitive and negative symptoms, and some early clinical trials have shown promising results at low doses (Keefe et al., 2015). The interaction between DA and ACh systems is also evident in recent studies from patients assessing DA system influence on ACh receptor expression where genotypic differences in COMT alter expression levels of muscarinic receptors, and these receptors are also found to be significantly reduced in PFC in a subset of patients (Scarr et al., 2009; Dean and Scarr, 2016). As ACh and dopamine systems show reciprocal connections and regulation, the selective reduction in M1 in PFC may be due to sensitivity of this region to changes in multiple neuromodulatory and catacholaminergic systems (Arnsten, 2015).

As ACh is critical for development of both sensory systems and higher order association cortices, the developmental nature of schizophrenia and the associated cognitive symptoms suggest a role for ACh dysfunction in the disease, at least in some patients (Reichenberg et al., 2010). Studies have shown changes in these systems following prenatal and/or adolescent tobacco exposure in humans, supporting a key role for ACh in childhood and adolescent brain development (Jacobsen et al., 2007a,b,c). Additionally, schizophrenic patients smoke tobacco at substantially higher rates than the general population, indicating a method of self-medication to strengthen dlPFC circuits critical for attention and working memory (Hughes et al., 1986). As the high affinity α4β2 nicotinic receptor also plays a role in PFC circuitry, treatments targeting nicotinic receptors may be a promising avenue for pharmacological development. The α4β2 receptor is also highly expressed in the nucleus accumbens and underlies the reinforcing and addictive properties of nicotine (Picciotto et al., 1998), indicating these receptors may not be optimal to target. Instead, pharmacological developments have targeted nicotinic α7 receptors, with mixed results (Freedman et al., 2008; Keefe et al., 2015; Haig et al., 2016). As there is a steep “inverted U” dose response with this mechanism, lower doses or lower affinity agonists may be needed to avoid nonspecific excitatory actions that are harmful to information processing (Arnsten and Wang, 2016). More recent clinical interest has also focused on muscarinic M1 receptors, as these are the most highly expressed in cortex, with lower subcortical expression, and are associated with SZ (Scarr et al., 2009). However, these compounds must be highly selective for the M1R to avoid toxic muscarinic e.g., M2 cardiac side effects (e.g., Freedman et al., 1993).

The cholinergic system has also long been implicated in Alzheimer's Disease (AD), since the finding that basal forebrain nuclei degenerate in AD (Whitehouse et al., 1982). As described above, the regions with the greatest pathological burden, the temporal lobes and hippocampal formation, as well as the PFC, receive strong basal forebrain cholinergic innervation and critically depend on ACh for optimal circuit function. This loss of cholinergic innervation provided one of the first early medication options to temporarily improve function in patients by giving acetylcholinesterase inhibitors to prolong ACh actions in the synapse (Rogers et al., 1998). Although these compounds are in widespread use, they provide only temporary relief and do not halt the underlying degeneration of association cortex. Cholinergic receptors have also been implicated in the pathology of AD, with evidence that β-amyloid_1−42_ binds with high affinity to the nicotinic α7 receptor (Wang et al., 2000), and that this receptor facilitates the accumulation of β-amyloid_1−42_ within neurons (Nagele et al., 2002). Thus, ACh receptors may actually have detrimental effects on AD pathology. The critical role for this receptor in recurrent dlPFC circuits, and the sensitivity of these circuits to insult due to a high level of recurrent excitatory connections, may thus be one mechanism behind the sensitivity of this region to degeneration in AD.

## Conclusion

While there is still much we do not understand about the precise function of ACh and its receptors in development and mature brain processing, it is evident ACh plays a critical role in cortical development and in higher cortical processing. ACh signaling plays a key role in amplifying incoming sensory signals, and is critical for higher order cognitive processes, permitting critical activation of PFC circuits underlying working memory. It appears to be particularly important for providing neural excitation under conditions when there is no or little sensory stimulation, e.g., during development of the visual system prior to eye opening, or in mature dlPFC microcircuits that need to maintain representations of events in the absence of sensory stimulation. The differences in cholinergic actions across primary sensory to high order association cortices is correlated with strength of attentional modulation, is tailored to the different functions of each cortical area, and exerts influence via actions at nicotinic and muscarinic receptors which show highly different expression patterns across cortical regions. The heaviest cholinergic innervation of limbic cortical areas is reminiscent of the pattern of degeneration in neuropsychiatric diseases with altered ACh levels or receptor function. The differences in PFC and V1 for ACh function may be useful in guiding development of future therapeutics for such disorders, using low-dose therapies to improve PFC circuit function without altering other brain regions and systems.

## Author contributions

AA and VG contributed to formation of review topic. AA and MW supported and guided VG in writing of review.

### Conflict of interest statement

The authors declare that the research was conducted in the absence of any commercial or financial relationships that could be construed as a potential conflict of interest.
